# HDAC Inhibition Modulates Cardiac PPARs and Fatty Acid Metabolism in Diabetic Cardiomyopathy

**DOI:** 10.1155/2016/5938740

**Published:** 2016-06-30

**Authors:** Ting-I Lee, Yu-Hsun Kao, Wen-Chin Tsai, Cheng-Chih Chung, Yao-Chang Chen, Yi-Jen Chen

**Affiliations:** ^1^Department of General Medicine, School of Medicine, College of Medicine, Taipei Medical University, Taipei 11031, Taiwan; ^2^Division of Endocrinology and Metabolism, Department of Internal Medicine, Wan Fang Hospital, Taipei Medical University, Taipei 11696, Taiwan; ^3^Graduate Institute of Clinical Medicine, College of Medicine, Taipei Medical University, Taipei 11031, Taiwan; ^4^Department of Medical Education and Research, Wan Fang Hospital, Taipei Medical University, Taipei 11696, Taiwan; ^5^Division of Cardiology, Tzu-Chi General Hospital, Institute of Medical Sciences, Tzu-Chi University, Hualien 97004, Taiwan; ^6^Division of Cardiovascular Medicine, Department of Internal Medicine, Wan Fang Hospital, Taipei Medical University, Taipei 11696, Taiwan; ^7^Department of Biomedical Engineering, National Defense Medical Center, Taipei 11490, Taiwan

## Abstract

Peroxisome proliferator-activated receptors (PPARs) regulate cardiac glucose and lipid homeostasis. Histone deacetylase (HDAC) inhibitor has anti-inflammatory effects which may play a key role in modulating PPARs and fatty acid metabolism. The aim of this study was to investigate whether HDAC inhibitor, MPT0E014, can modulate myocardial PPARs, inflammation, and fatty acid metabolism in diabetes mellitus (DM) cardiomyopathy. Electrocardiography, echocardiography, and western blotting were used to evaluate the electrophysiological activity, cardiac structure, fatty acid metabolism, inflammation, and PPAR isoform expressions in the control and streptozotocin-nicotinamide-induced DM rats with or without MPT0E014. Compared to control, DM and MPT0E014-treated DM rats had elevated blood glucose levels and lower body weights. However, MPT0E014-treated DM and control rats had smaller left ventricular end-diastolic diameter and shorter QT interval than DM rats. The control and MPT0E014-treated DM rats had greater cardiac PPAR-*α* and PPAR-*δ* protein expressions, but less cardiac PPAR-*γ* than DM rats. Moreover, control and MPT0E014-treated DM rats had lower concentrations of 5′ adenosine monophosphate-activated protein kinase 2*α*, PPAR-*γ* coactivator 1*α*, phosphorylated acetyl CoA carboxylase, cluster of differentiation 36, diacylglycerol acyltransferase 1 (DGAT1), DGAT2, tumor necrosis factor-*α*, and interleukin-6 protein than DM rats. HDAC inhibition significantly attenuated DM cardiomyopathy through modulation of cardiac PPARS, fatty acid metabolism, and proinflammatory cytokines.

## 1. Introduction

Diabetes mellitus (DM) is a complex, chronic illness in which there is an absolute or relative lack of insulin. DM affected 285 million adults worldwide in 2010, and this number is expected to reach 439 million by 2030 [1]. Cardiovascular complications are the leading cause of DM-related morbidity and mortality [2, 3]. DM cardiomyopathy adversely affects the DM outcomes, and it is generated from enhanced oxidative stress, inflammation, fibrosis, metabolic abnormalities, and vascular diseases [4, 5]. Clinical and animal studies showed that hyperglycemia causes metabolic derangement in DM cardiomyocytes [6, 7]. Intramyocellular free fatty acids (FFA) accumulation due to fixed myocardial substrate utilization causes cardiac lipotoxicity, which leads to myocardial dysfunction in DM [8]. Although blood glucose control plays a vital role in treating DM, the current management of DM cardiomyopathy remains insufficient.

The inhibition of histone deacetylases (HDACs) has been widely studied in targeting cancers. Moreover, HDAC inhibition may modulate cardiovascular diseases through the regulation of oxidative stress, inflammation, the renin-angiotensin system, and metabolism [9–11]. Deletion of cardiac HDAC3 results in cardiac hypertrophy and metabolic derangements including upregulation of genes involved in fatty acid uptake and oxidation as well as attenuation of glucose uptake [12]. DM is associated with increased inflammation and oxidative stress, which may result in dysregulated cardiac metabolism [6, 13, 14]. Studies demonstrated that elevated FFA oxidation and impaired utilization of glucose may have detrimental effects on DM cardiac function [15–18]. HDAC inhibition has a significant effect in suppressing oxidative stress and inflammation in a hyperglycemic condition [19]. HDAC activity is upregulated in cardiomyopathy [11, 20]. However, the metabolic roles of HDAC inhibition in the DM hearts have not yet been elucidated. It remains unclear whether HDAC inhibition regulates DM cardiomyopathy by attenuating the fatty acid metabolism disorders. Furthermore, peroxisome proliferator-activated receptors- (PPARs-) *α*, *γ*, and *δ* form heterodimers with the retinoid X receptor (RXR) and regulate energy utilization and storage [21]. The coordinated effects of PPARs and RXR help regulate inflammation and atherosclerosis [22]. Our previous study found that DM can modulate PPARs in the cardiomyocytes [17]. Moreover, the unliganded PPAR-*γ* heterodimeric complex is associated with a multicomponent corepressor complex that contains HDAC activity. After binding to the PPAR-*γ* ligand, the corepressor complex is dismissed, and the coactivator complex that possesses histone acetylase activity is recruited to the PPAR*γ*/RXR heterodimer. This leads to chromatin remodeling, which facilitates active transcription [23]. In addition, PPAR-*δ* represses the transcriptional activity of PPAR-*α* and PPAR-*γ* through binding to PPAR response element and its associated repressor complex and HDAC [24]. However, the regulatory effects of the HDAC inhibitor on cardiac PPAR isoform expressions in DM cardiomyocytes remain unclear. Therefore, the purpose of this study was to investigate whether HDAC inhibitor, MPT0E014, can modulate PPARs, fatty acid metabolism, and inflammation in DM hearts.

## 2. Materials and Methods

### 2.1. Animal, Blood Sampling, and Tissue Preparations

This investigation conformed to the institutional* Guide for the Care and Use of Laboratory Animals* and the* Guide for the Care and Use of Laboratory Animals* published by the US National Institutes of Health (NIH Publication number 85-23, revised 1996) and was approved by the Institutional Animal Care and Use Committee of Taipei Medical University (LAC-2014-0237). Rats were housed in standard environmental conditions and maintained on commercial rat chow and tap water ad libitum. To induce DM, 10-week-old male Wistar rats (~335 g ± 4.5) received nicotinamide (150 mg/kg, Sigma-Aldrich, St. Louis, MO, USA) intraperitoneally 15 min before a single intraperitoneal injection of streptozotocin (65 mg/kg STZ, Sigma) after a 10-hour overnight starvation [25]. DM was diagnosed with high fasting plasma glucose (≥15 mmol/L) as measured with a glucometer (Ascensia Elite, Bayer Health Care, Mishawaka, IN, USA) [18, 26]. At 12 weeks of age, the rats were grouped into control, DM, and MPT0E014-treated DM. MPT0E014 (a pan-HDAC inhibitor [10], 50 mg/kg in 50% polyethylene glycol 400 and 0.25% carboxymethyl cellulose) [20] or vehicle was given once daily for 7 days by oral gavage in the experimental rats. The rats were anesthetized intraperitoneally with sodium pentobarbital (100 mg/kg) and sacrificed at 13 weeks of age. Body weights were measured, and blood samples were extracted prior to euthanasia. Transverse tissue pieces from the ventricles were snap-frozen in liquid nitrogen for protein isolation. Fasting serum total cholesterol, triglyceride, and high-density lipoprotein-cholesterol were obtained by SPOTCHEM analyzer (Minami-Ku, Kyoto, Japan) using SPOTCHEM II Inorganic Phosphorus reagent strips. Serum FFA was measured with Rat ELISA kit (Sigma), and serum fasting insulin was measured by using a Mercodia Ultrasensitive Rat Insulin ELISA (Mercodia AB, Sweden).

### 2.2. Echocardiographic and Electrocardiographic Measurements

Transthoracic echocardiography was performed using the Vivid I ultrasound cardiovascular system (GE Healthcare, Haifa, Israel) under isoflurane anesthesia (5% for induction and 2% for maintenance) at 10 and 13 weeks of age in the control and also in the DM rats with and without MPT0E014 administration. M-Mode tracing of the left ventricle (LV) was used to measure the following cardiac structures: the LV end-diastolic diameter (LVEDd), LV end-systolic diameter (LVESd), interventricular septal thickness in diastole (IVSd), end-diastolic volume (EDV), end-systolic volume (ESV), fractional shortening (FS), and ejection fraction (EF) [10].

Electrocardiograms (ECGs) were performed at 10 and 13 weeks of age and were recorded from standard lead II limb leads via a bioamplifier (ADInstruments, Castle Hill, Australia), connected to a polygraph recorder (ML 845 Powerlab, ADInstruments). Results were continuously displayed throughout the experiment in the control as well as the DM rats with and without MPT0E014 treatment.

### 2.3. Western Blot Analysis

Equal amounts of proteins (40 *μ*g) were resolved by sodium dodecylsulfate polyacrylamide gel electrophoresis (SDS-PAGE) followed by electrophoretic transfer of proteins onto nitrocellulose membranes. Blots were probed with antibodies against PPAR-*α* (Santa Cruz Biotechnology, Santa Cruz, CA, USA), PPAR-*γ* (Santa Cruz Biotechnology), PPAR-*δ* (Affinity Bio Reagent, Golden, CO, USA), tumor necrosis factor- (TNF-) *α* (AbDSerotec, MorphoSys UK, Oxford, UK), interleukin- (IL-) 6 (Bender MedSystems, Vienna, Austria), PPAR-*γ* coactivator- (PGC-) 1*α* (Abcam, Cambridge, UK), 5′ adenosine monophosphate-activated protein kinase 2*α* (AMPK2*α*) (Cell Signaling, Beverly, MA, USA), phosphorylated acetyl coenzyme A carboxylase (pACC) (Millipore, St. Louis, MO, USA), diacylglycerol acyltransferase 1 (DGAT1) (Abcam), DGAT2 (Abcam), cluster of differentiation 36 (CD36) (Abcam), phosphorylated AMPK2*α* (pAMPK2*α*) (Millipore), Akt (Cell Signaling), phosphorylated Akt (pAkt) (Cell Signaling), and secondary antibodies conjugated with horseradish peroxidase (HRP; Leinco Technology, St. Louis, MO, USA). Bound antibodies were detected with an enhanced chemiluminescence detection system (Millipore) and analyzed with AlphaEaseFC software (Alpha Innotech, San Leandro, CA, USA). Targeted bands were normalized to cardiac glyceraldehyde 3-phosphate dehydrogenase (GAPDH) (Sigma-Aldrich) to confirm equal protein loading.

### 2.4. Statistical Analysis

All quantitative data are expressed as mean ± standard error of the mean (SEM). Statistical significance between different groups was determined by an unpaired *t*-test or a one-way analysis of variance (ANOVA) with Tukey's test for multiple comparisons as appropriate. A value of *P* < 0.05 was considered statistically significant.

## 3. Results

### 3.1. Blood Glucose, Cardiac Structure, and ECG of Control, DM, and MPT0E014-Treated DM Rats

**Figure 1:**
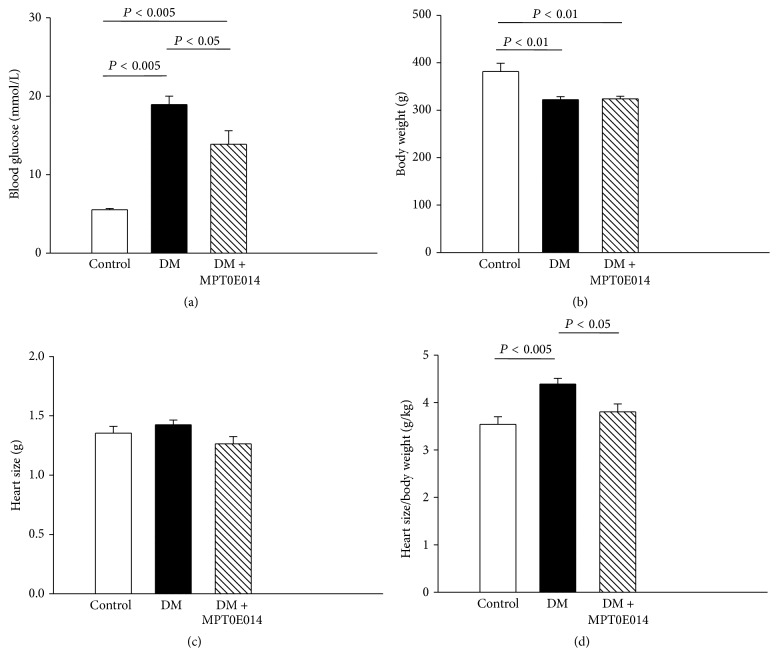
Fasting blood glucose level and heart size for control, diabetes mellitus (DM), and MPT0E014-treated DM (DM + MPT0E014) rats. Average data of (a) fasting blood glucose level, (b) body weight, (c) heart size, and (d) heart-size-to-body-weight ratio at 13 weeks of age in control (*n* = 5), DM (*n* = 5), and DM + MPT0E014 (*n* = 5) rats. Values are expressed as the mean ± SEM.

DM and MPT0E014-treated DM rats at 13 weeks of age had elevated blood glucose levels as compared to control rats and to their own respective baselines (Figure 1(a)). In addition, the MPT0E014-treated DM rats had modestly lower blood glucose levels than DM rats. The DM and MPT0E014-treated DM rats had lower body weights than control rats (Figure 1(b)). As compared to control rats, we found that the serum insulin levels of DM and MPT0E014-treated DM rats were decreased to a similar extent (Table 1). The absolute weights of the hearts were similar among control, DM, and MPT0E014-treated DM rats (Figure 1(c)). In addition, the heart-to-body weight ratios were greater in DM rats compared to control rats and MPT0E014-treated DM rats (Figure 1(d)).

**Figure 2:**
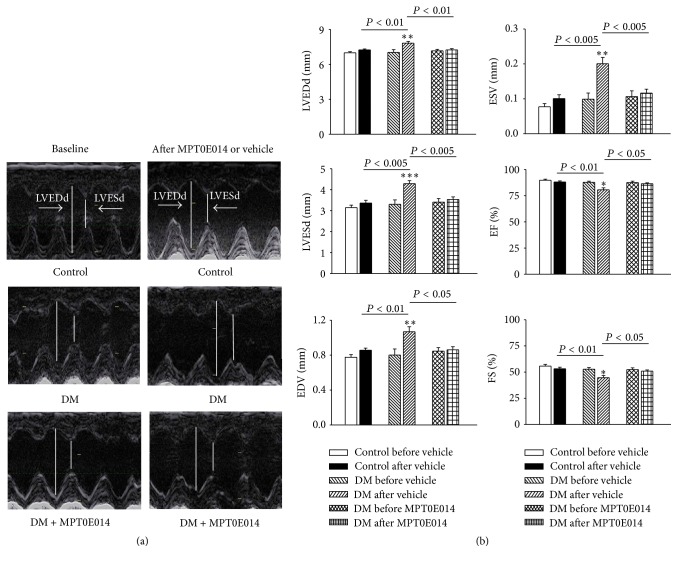
Effects of the histone deacetylase (HDAC) inhibitor, MPT0E014, on echocardiograms of control, diabetes mellitus (DM), and MPT0E014-treated DM (DM + MPT0E014) rats. (a) Representative echocardiographic images of control, DM, and DM + MPT0E014 rats. (b) Average data of the left ventricular end-diastolic diameter (LVEDd), left ventricular end-sytolic diameter (LVESd), fractional shortening (FS), and ejection fraction (EF) in control (*n* = 6), DM (*n* = 6), and DM + MPT0E014 (*n* = 6) rats. ^*∗*^ *P* < 0.05 versus the DM group, ^*∗∗*^ *P* < 0.01 versus the DM group, and ^*∗∗∗*^ *P* < 0.005 versus the DM group, before and after treatment with the vehicle.

**Figure 3:**
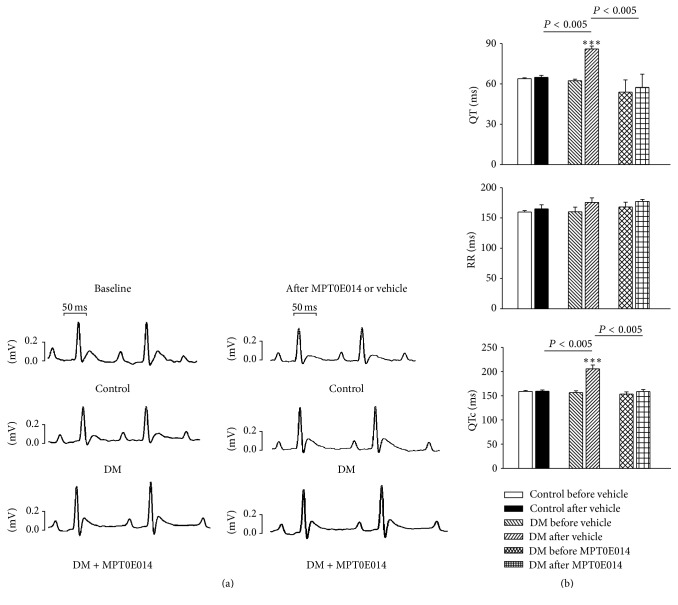
Electrocardiographic changes in control, diabetes mellitus (DM), and MPT0E014-treated DM (DM + MPT0E014) rats. (a) Representative electrocardiographic tracings of control, DM, and DM + MPT0E014 rats. (b) Mean electrocardiographic values of the control (*n* = 7), DM (*n* = 7), and DM + MPT0E014 (*n* = 7) rats. ^*∗∗∗*^ *P* < 0.005 versus the DM group, before and after treatment with the vehicle.

Figure 2 depicts echocardiograms of control, DM, and MPT0E014-treated DM rats before and after treatment. The DM rats had increased LVEDd, LVESd, EDV, and ESV values and lower EF and FS values compared to the control and MPT0E014-treated DM rats. Moreover, the DM rats had longer QT interval and corrected QT (QTc) intervals than either the control or MPT0E014-treated DM rats. However, the RR intervals were similar among the three groups (Figure 3).

### 3.2. Myocardial Fatty Acid Metabolic Pathway and Serum Lipid Parameters in the Control, DM, and MPT0E014-Treated DM Rats

**Figure 4:**
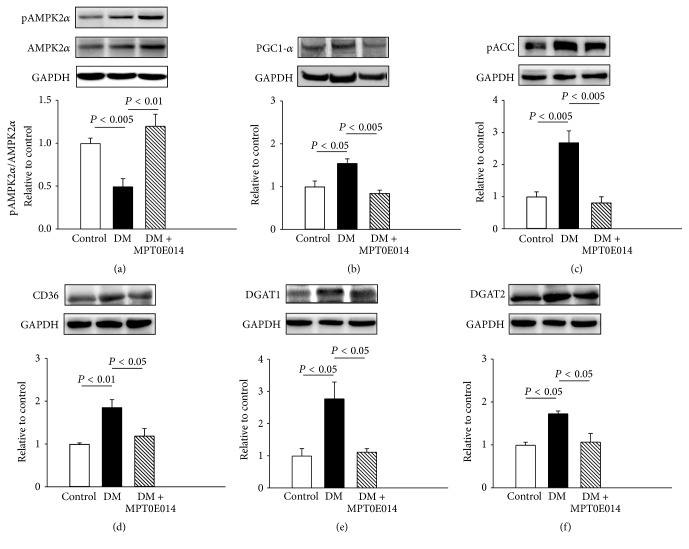
Cardiac fatty acid metabolic proteins in control, diabetes mellitus (DM), and MPT0E014-treated DM (DM + MPT0E014) rats. Representative immunoblots and average data of (a) ratio of phosphorylated 5′ adenosine monophosphate-activated protein kinase 2*α* (pAMPK2*α*) to total AMPK2*α*, (b) peroxisome proliferator-activated receptor- (PPAR-) *γ* coactivator- (PGC-) 1*α*, (c) phosphorylated acetyl coenzyme A carboxylase (pACC), (d) cluster of differentiation 36 (CD36), (e) diacylglycerol acyltransferase 1 (DGAT1), and (f) DGAT2 from control (*n* = 4), DM (*n* = 4), and DM + MPT0E014 (*n* = 4) rats. Densitometry was normalized to glyceraldehyde 3-phosphate dehydrogenase (GAPDH) as an internal control.

As shown in Figure 4 (Figure 4(a)), compared to those in the control rats, DM hearts showed a lower ratio of pAMPK2*α* to total AMPK2*α* and greater expressions of PGC-1*α* and pACC (Figures 4(b) and 4(c)), which were attenuated in DM rats treated with MPT0E014. Cardiac CD36 protein expression was also 0.86-fold higher in DM hearts than in the healthy hearts (Figure 4(d)), and this was attenuated in MPT0E014-treated DM hearts. Moreover, compared to control hearts, cardiac DGAT1 and DGAT2 protein levels were, respectively, upregulated by 1.8-fold and 0.73-fold in DM hearts. However, MPT0E014-treated DM and control hearts had similar expressions of DGAT1 and DGAT2 proteins (Figures 4(e) and 4(f)).

At 13 weeks of age, total cholesterol, triglycerides, and FFA were higher in the DM rats compared to the healthy rats and were attenuated by treatment with MPT0E014 (Table 1).

### 3.3. PPAR Isoforms, Proinflammatory Cytokines, and pAKT in the Control, DM, and MPT0E014-Treated DM Hearts

**Figure 5:**
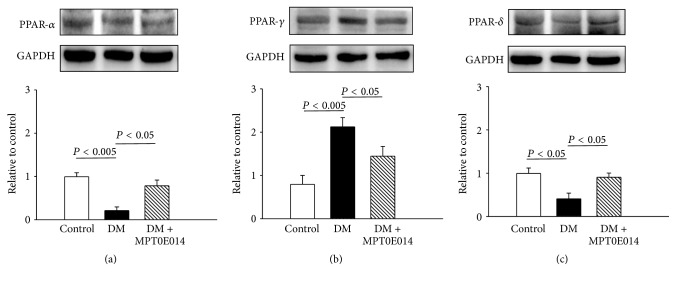
Cardiac peroxisome proliferator-activated receptor (PPAR) proteins in control, diabetes mellitus (DM), and MPT0E014-treated DM (DM + MPT0E014) rats. Cardiac PPAR-*α* and PPAR-*δ* protein expressions significantly decreased in DM (*n* = 4) compared to control (*n* = 4) and DM + MPT0E014 (*n* = 4) rats. Cardiac PPAR-*γ* protein expressions were enhanced in DM (*n* = 4) compared to control (*n* = 4) and DM + MPT0E014 (*n* = 4) rats. Representative immunoblots and average data of (a) PPAR-*α*, (b) PPAR-*γ*, and (c) PPAR-*δ* in different groups. Densitometry was normalized to glyceraldehyde 3-phosphate dehydrogenase (GAPDH) as an internal control.

DM hearts were associated with a 0.78-fold lower PPAR-*α* protein level compared to control hearts (Figure 5(a)). However, control and MPT0E014-treated DM hearts showed similar expressions of the PPAR-*α* protein. DM hearts had 0.99-fold higher PPAR-*γ* expression (Figure 5(b)) and 0.62-fold lower PPAR-*δ* expression than control hearts (Figure 5(c)), but there were similar PPAR-*γ* and PPAR-*δ* expressions between control and MPT0E014-treated DM hearts (Figures 5(b) and 5(c)).

**Figure 6:**
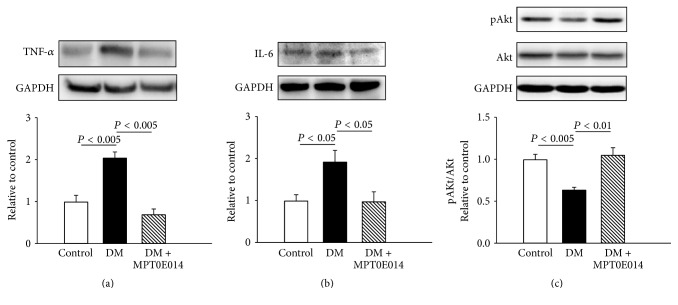
Cardiac inflammatory proteins and ratio of phosphorylated Akt to total Akt in control, diabetes mellitus (DM), and MPT0E014-treated DM (DM + MPT0E014) rats. Cardiac tumor necrosis factor- (TNF-) *α* and interleukin- (IL-) 6 protein levels were significantly higher in DM rats (*n* = 4) compared to control (*n* = 4) and DM + MPT0E014 (*n* = 4) rats. Representative immunoblots and average data of (a) TNF-*α*, (b) IL-6, and (c) ratio of pAkt to total Akt in different groups. Densitometry was normalized to glyceraldehyde 3-phosphate dehydrogenase (GAPDH) as an internal control.

Compared to control hearts, DM hearts were associated with greater expressions of TNF-*α* and IL-6 than control hearts by 1-fold and 0.77-fold, respectively (Figures 6(a) and 6(b)). However, similar levels of TNF-*α* and IL-6 proteins were found between the control and MPT0E014-treated DM hearts. Additionally, DM hearts had a lesser expression of pAKT to total Akt ratio than control hearts, which was attenuated in DM hearts treated with MPT0E014 (Figure 6(c)).

## 4. Discussion

DM is associated with a proinflammatory state and increased tissue concentrations of cytokines [27]. Emerging data demonstrated that HDAC inhibitors have promising anti-inflammatory properties [28]. Thus, HDAC inhibitors may have a cardioprotective potential in DM cardiomyopathy through their anti-inflammatory effects. HDAC inhibition has been associated with improvements in insulin signaling [29]. This study showed that DM rats with and without HDAC inhibition had similar plasma insulin levels. However, the treatment of MPT0E014 reversed the decrease of pAKT in the DM hearts. These findings suggest that inhibition of HDAC may improve insulin signaling and result in improved cardiac function and metabolic homeostasis in DM hearts. Furthermore, we have demonstrated that myocardial inflammatory cytokines TNF-*α* and IL-6 protein expressions were elevated congruently with the increased blood glucose levels in the DM group. Since MPT0E014 only minimally reduces blood glucose level, the significant reductions in myocardial TNF-*α* and IL-6 proteins in MPT0E014-treated DM rats suggest that the HDAC inhibitor, MPT0E014, may serve as an anti-inflammatory agent for DM cardiomyopathy independent of glucose control at least in part. Thus, the underlying mechanism for the blood glucose lowering effect of MPT0E014 in DM cardiomyopathy may be caused by improving insulin sensitivity that was attributed to an amelioration of inflammation.

DM is also associated with cardiac lipid accumulation. Myocardial lipotoxicity may have resulted from increased lipid uptake in the cardiomyocytes which may result in adverse effects on the myocardial function. Similar to previous investigations [13, 30], this study found that DM rats had abnormal myocardial fatty acid metabolic signals. AMPK is an important mediator of glucose metabolism in the myocardium [31], and it plays an important role in regulating exchange of energy metabolism during cellular stress [32]. Moreover, since AMPK also facilitates fatty acid utilization through its control of ACC, the activation of AMPK2*α* in the DM hearts likely increased fatty acid oxidation through phosphorylation and inhibition of ACC [33]. In addition, AMPK stimulates PGC-1*α* expression and directly augments its activity through AMPK phosphorylation [34]. PGC-1*α* plays an important role in regulating fatty acid *β*-oxidation and excess myocardial lipid accumulation that might worsen cardiac lipid and glucose utilization and the energy balance [35]. AMPK requires PGC-1*α* activity in order to modulate expressions of several key players in mitochondrial and glucose metabolism [34]. Thus, PGC-1*α* plays a vital role in the function of AMPK as a response to energy during stress situations.

Elevation of the fatty acid transporter protein, CD36, in DM hearts may facilitate intracellular fatty acid uptake, as evidenced by elevated AMPK2*α* and pACC protein levels. AMPK was also implicated in fatty acid delivery to cardiomyocytes through its regulation of the fatty acid transporter, CD36 [36]. Although Montgomery et al. reported that deletion of cardiac HDAC3 did not significantly change CD36 in healthy hearts [12], we found that HDAC inhibition attenuated the DM-induced elevation of CD36. Since AMPK2*α* and pACC protein levels were reversed after treatment with the HDAC inhibitor, MPT0E014, in the DM rats, our findings suggest that HDAC inhibitors may act as a key regulator of fatty acid substrates in the myocardium. The mechanism of lipotoxicity in DM cardiomyopathy not only arises from the accumulation of triacylglycerol per se but is also a result of increased availability of lipid intermediates such as diacylglycerol. DGAT exists in two isoforms of DGAT1 and DGAT2 that catalyze the final step in the biosynthesis of triglycerides [37]. DGAT1 is related to the kinetic rate of triglyceride absorption from the gastrointestinal tract and energy expenditure [38]. DGAT2 appears to be dominant in controlling in vivo triglyceride homeostasis [39]. Both DGAT1 and DGAT2 were found to be elevated in our DM hearts. The excess supply of fatty acids in DM hearts may have generated reactive oxidative stress that could also have caused significant myocardial damage in DM cardiomyopathy.

We found a significantly dilated LV chamber with a lower EF and a shorter FS in DM hearts, as in previous investigations [40, 41]. In addition, the DM hearts were associated with a larger heart-size-to-body-weight ratio as well as prolonged QT and QTc intervals. Previous investigations showed that normalizing fatty acid metabolism in DM hearts reverses impaired myocardial contractility [42, 43]. Interestingly, together with the reversal in the ratio of pAMPK2*α* to total AMPK2*α* and other myocardial fatty acid regulators, we also found significant improvements in myocardial systolic and diastolic functions with shortening of the QT and QTc intervals and a decreased heart-size-to-body-weight ratio in MPT0E014-treated DM rats. These findings suggest that the improvement in cardiac function is associated with sufficient energy after HDAC inhibition. HDAC inhibitor, MPT0E014, may have cardioprotective potential in DM cardiomyopathy.

Similar to our previous studies [13, 17], there were significant declines in both PPAR-*α* and PPAR-*δ* proteins and an increase in myocardial PPAR-*γ* protein levels in our DM hearts despite an increase in fatty acid oxidation, suggesting a compensatory response in preserving the contractile function induced by proinflammatory cytokines during hyperglycemia [44]. Consistent with previous studies that focused on the early stages of DM, we found that cardiac PPAR-*α* protein levels were downregulated after 3 weeks of DM [45–48]. In addition, cardiac fatty acid metabolism was increased in the patients with type 1 DM [49], which is similar to the findings in our studies. However, several other studies have reported that cardiac PPAR-*α* is increased in DM animal models [50, 51]. The disparities between these studies may be due to different experimental conditions such as the genetic background, animal species, and severity or duration of DM.

For the first time, we demonstrated that the HDAC inhibitor, MPT0E014, reversed the hyperglycemic effects on cardiac PPARs in DM hearts. Since inflammation can regulate expressions of PPAR isoforms [17, 18, 44], the effect of MPT0E014 on cardiac PPARs may have resulted from its anti-inflammatory activity. Moreover, MPT0E014 may modulate cardiac metabolism through its effects on PPARs and inflammatory cytokines to diminish the accumulation of fatty acids in DM hearts.

### 4.1. Study Limitations

These data should be interpreted with caution because of limitations of this study. Although the HDAC inhibitor, MPT0E014, was found to change cardiac fatty acid metabolism, it would have been useful in this study design to elucidate the direct effects of MPT0E014 in the cardiac metabolism by directly measuring carbohydrate and fatty acid utilization. Moreover, the downstream signaling pathway underlying the activity of MPT0E014 on myocardial inflammatory cytokines and PPAR expressions was not fully elucidated in our study. The direct impact of MPT0E014 on PPAR gene expressions may warrant further investigation.

## 5. Conclusions

MPT0E014 improves the cardiovascular changes in a DM rat model which may have been caused by its effects on cardiac PPARs, fatty acid regulation, and proinflammatory cytokines.

## Figures and Tables

**Table 1:** Lipid parameters of Wistar, diabetes mellitus (DM), and MPT0E014-treated DM rats.

	WKY	DM	DM + MPT0E104
Cholesterol (mmol/L)	1.4 ± 0.1^b^	2.1 ± 0.1^a,c^	1.5 ± 0.1^b^
Triglyceride (mmol/L)	1.1 ± 0.2^b^	3.0 ± 0.5^a,c^	1.0 ± 0.5^b^
HDL-C (mmol/L)	0.3 ± 0.3	0.4 ± 0.4	0.4 ± 0.4
Free fatty acid (*μ*mol/L)	17.5 ± 1.7^b^	30.9 ± 3.8^a,c^	16.8 ± 2.9^b^
Insulin (pmol/L)	141.2 ± 28.3^b,c^	55.0 ± 10.8^a^	67.2 ± 13.2^a^

HDL-C: high-density lipoprotein-cholesterol. Values are expressed as the mean ± SEM, *n* = 5.  ^a^ *P* < 0.05 versus Wistar rats, ^b^ *P* < 0.05 versus DM rats, and ^c^ *P* < 0.05 versus MPT0E014-treated DM rats.

## References

[1] Shaw J. E., Sicree R. A., Zimmet P. Z. (2010). Global estimates of the prevalence of diabetes for 2010 and 2030. *Diabetes Research and Clinical Practice*.

[2] Garcia M. J., McNamara P. M., Gordon T., Kannell W. B. (1974). Morbidity and mortality in diabetics in the Framingham population. Sixteen year follow-up study. *Diabetes*.

[3] Giles T. D. (2003). The patient with diabetes mellitus and heart failure: at-risk issues. *The American Journal of Medicine*.

[4] Giacco F., Brownlee M. (2010). Oxidative stress and diabetic complications. *Circulation Research*.

[5] Pillarisetti S., Saxena U. (2004). Role of oxidative stress and inflammation in the origin of Type 2 diabetes—a paradigm shift. *Expert Opinion on Therapeutic Targets*.

[6] Fuentes-Antrás J., Picatoste B., Ramírez E., Egido J., Tuñón J., Lorenzo Ó. (2015). Targeting metabolic disturbance in the diabetic heart. *Cardiovascular Diabetology*.

[7] Bugger H., Abel E. D. (2014). Molecular mechanisms of diabetic cardiomyopathy. *Diabetologia*.

[8] Stratmann B., Tschoepe D. (2011). Heart in diabetes: not only a macrovascular disease. *Diabetes Care*.

[9] Christensen D. P., Dahllöf M., Lundh M. (2011). Histone deacetylase (HDAC) inhibition as a novel treatment for diabetes mellitus. *Molecular Medicine*.

[10] Kao Y.-H., Liou J.-P., Chung C.-C. (2013). Histone deacetylase inhibition improved cardiac functions with direct antifibrotic activity in heart failure. *International Journal of Cardiology*.

[11] Chen Y., Du J., Zhao Y. T. (2015). Histone deacetylase (HDAC) inhibition improves myocardial function and prevents cardiac remodeling in diabetic mice. *Cardiovascular Diabetology*.

[12] Montgomery R. L., Potthoff M. J., Haberland M. (2008). Maintenance of cardiac energy metabolism by histone deacetylase 3 in mice. *The Journal of Clinical Investigation*.

[13] Lee T.-I., Kao Y.-H., Chen Y.-C., Tsai W.-C., Chung C.-C., Chen Y.-J. (2014). Cardiac metabolism, inflammation, and peroxisome proliferator-activated receptors modulated by 1,25-dihydroxyvitamin D3 in diabetic rats. *International Journal of Cardiology*.

[14] Aronson D., Rayfield E. J. (2002). How hyperglycemia promotes atherosclerosis: molecular mechanisms. *Cardiovascular Diabetology*.

[15] Finck B. N. (2007). The PPAR regulatory system in cardiac physiology and disease. *Cardiovascular Research*.

[16] Saunders J., Mathewkutty S., Drazner M. H., McGuire D. K. (2008). Cardiomyopathy in type 2 diabetes: update on pathophysiological mechanisms. *Herz*.

[17] Lee T.-I., Kao Y.-H., Chen Y.-C., Pan N.-H., Chen Y.-J. (2010). Oxidative stress and inflammation modulate peroxisome proliferator-activated receptors with regional discrepancy in diabetic heart. *European Journal of Clinical Investigation*.

[18] Lee T.-I., Kao Y.-H., Chen Y.-C., Pan N.-H., Lin Y.-K., Chen Y.-J. (2011). Cardiac peroxisome-proliferator-activated receptor expression in hypertension co-existing with diabetes. *Clinical Science*.

[19] Miao F., Gonzalo I. G., Lanting L., Natarajan R. (2004). In vivo chromatin remodeling events leading to inflammatory gene transcription under diabetic conditions. *The Journal of Biological Chemistry*.

[20] Lkhagva B., Lin Y.-K., Kao Y.-H. (2015). Novel histone deacetylase inhibitor modulates cardiac peroxisome proliferator-activated receptors and inflammatory cytokines in heart failure. *Pharmacology*.

[21] Chen L., Jia Z., Yang G. (2014). PPARs and metabolic syndrome. *PPAR Research*.

[22] Brown J. D., Plutzky J. (2007). Peroxisome proliferator-activated receptors as transcriptional nodal points and therapeutic targets. *Circulation*.

[23] Glass C. K., Rosenfeld M. G. (2000). The coregulator exchange in transcriptional functions of nuclear receptors. *Genes & Development*.

[24] Shi Y., Hon M., Evans R. M. (2002). The peroxisome proliferator-activated receptor *δ*, an integrator of transcriptional repression and nuclear receptor signaling. *Proceedings of the National Academy of Sciences of the United States of America*.

[25] Masiello P., Broca C., Gross R. (1998). Experimental NIDDM: development of a new model in adult rats administered streptozotocin and nicotinamide. *Diabetes*.

[26] Lee T.-I., Chen Y.-C., Kao Y.-H., Hsiao F.-C., Lin Y.-K., Chen Y.-J. (2013). Rosiglitazone induces arrhythmogenesis in diabetic hypertensive rats with calcium handling alteration. *International Journal of Cardiology*.

[27] Diamant M., Lamb H. J., Smit J. W. A., De Roos A., Heine R. J. (2005). Diabetic cardiomyopathy in uncomplicated type 2 diabetes is associated with the metabolic syndrome and systemic inflammation. *Diabetologia*.

[28] Halili M. A., Andrews M. R., Sweet M. J., Fairlie D. P. (2009). Histone deacetylase inhibitors in inflammatory disease. *Current Topics in Medicinal Chemistry*.

[29] Galmozzi A., Mitro N., Ferrari A. (2013). Inhibition of class i histone deacetylases unveils a mitochondrial signature and enhances oxidative metabolism in skeletal muscle and adipose tissue. *Diabetes*.

[30] Guo Z., Xia Z., Yuen V. G., McNeill J. H. (2007). Cardiac expression of adiponectin and its receptors in streptozotocin-induced diabetic rats. *Metabolism: Clinical and Experimental*.

[31] Rutter G. A., Da Silva Xavier G., Leclerc I. (2003). Roles of 5′-AMP-activated protein kinase (AMPK) in mammalian glucose homoeostasis. *Biochemical Journal*.

[32] Hardie D. G. (2003). Minireview: the AMP-activated protein kinase cascade: the key sensor of cellular energy status. *Endocrinology*.

[33] Kewalramani G., An D., Kim M. S. (2007). AMPK control of myocardial fatty acid metabolism fluctuates with the intensity of insulin-deficient diabetes. *Journal of Molecular and Cellular Cardiology*.

[34] Jäer S., Handschin C., St-Pierre J., Spiegelman B. M. (2007). AMP-activated protein kinase (AMPK) action in skeletal muscle via direct phosphorylation of PGC-1*α*. *Proceedings of the National Academy of Sciences of the United States of America*.

[35] Potthoff M. J., Inagaki T., Satapati S. (2009). FGF21 induces PGC-1*α* and regulates carbohydrate and fatty acid metabolism during the adaptive starvation response. *Proceedings of the National Academy of Sciences of the United States of America*.

[36] Luiken J. J. F. P., Coort S. L. M., Willems J. (2003). Contraction-induced fatty acid translocase/CD36 translocation in rat cardiac myocytes is mediated through AMP-activated protein kinase signaling. *Diabetes*.

[37] Shi Y., Cheng D. (2009). Beyond triglyceride synthesis: the dynamic functional roles of MGAT and DGAT enzymes in energy metabolism. *American Journal of Physiology—Endocrinology and Metabolism*.

[38] Yen C.-L. E., Stone S. J., Koliwad S., Harris C., Farese R. V. (2008). Thematic review series: glycerolipids. DGAT enzymes and triacylglycerol biosynthesis. *Journal of Lipid Research*.

[39] Stone S. J., Myers H. M., Watkins S. M. (2004). Lipopenia and skin barrier abnormalities in DGAT2-deficient mice. *Journal of Biological Chemistry*.

[40] Joffe I. I., Travers K. E., Perreault-Micale C. L. (1999). Abnormal cardiac function in the streptozotocin-induced, non-insulin-dependent diabetic rat: noninvasive assessment with Doppler echocardiography and contribution of the nitric oxide pathway. *Journal of the American College of Cardiology*.

[41] D'Amico M., Marfella R., Nappo F. (2001). High glucose induces ventricular instability and increases vasomotor tone in rats. *Diabetologia*.

[42] Wall S. R., Lopaschuk G. D. (1989). Glucose oxidation rates in fatty acid-perfused isolated working hearts from diabetic rats. *Biochimica et Biophysica Acta*.

[43] Chatham J. C., Forder J. R. (1997). Relationship between cardiac function and substrate oxidation in hearts of diabetic rats. *American Journal of Physiology—Heart and Circulatory Physiology*.

[44] Lee T.-I., Kao Y.-H., Chen Y.-C., Chen Y.-J. (2009). Proinflammatory cytokine and ligands modulate cardiac peroxisome proliferator-activated receptors. *European Journal of Clinical Investigation*.

[45] Bugger H., Dong C., Riehle C. (2009). Tissue-specific remodeling of the mitochondrial proteome in type 1 diabetic akita mice. *Diabetes*.

[46] Buchanan J., Mazumder P. K., Hu P. (2005). Reduced cardiac efficiency and altered substrate metabolism precedes the onset of hyperglycemia and contractile dysfunction in two mouse models of insulin resistance and obesity. *Endocrinology*.

[47] Rame J. E., Barouch L. A., Sack M. N. (2011). Caloric restriction in leptin deficiency does not correct myocardial steatosis: failure to normalize PPAR*α*/PGC1*α* and thermogenic glycerolipid/fatty acid cycling. *Physiological Genomics*.

[48] Drosatos K., Pollak N. M., Pol C. J. (2016). Cardiac myocyte KLF5 regulates *Ppara* expression and cardiac function. *Circulation Research*.

[49] Herrero P., Peterson L. R., McGill J. B. (2006). Increased myocardial fatty acid metabolism in patients with type 1 diabetes mellitus. *Journal of the American College of Cardiology*.

[50] Finck B. N., Lehman J. J., Leone T. C. (2002). The cardiac phenotype induced by PPAR*α* overexpression mimics that caused by diabetes mellitus. *The Journal of Clinical Investigation*.

[51] Banke N. H., Lewandowski E. D. (2015). Impaired cytosolic NADH shuttling and elevated UCP3 contribute to inefficient citric acid cycle flux support of postischemic cardiac work in diabetic hearts. *Journal of Molecular and Cellular Cardiology*.

